# Supplementary material for: Public investments in the clinical development of bedaquiline
Source: PLoS One. 2020 Sep 18;15(9):e0239118. doi: 10.1371/journal.pone.0239118 (PMC7500616; doi:10.1371/journal.pone.0239118)
Supplement: S1 File — (PDF) [file pone.0239118.s001.pdf]

# Appendix

## Table of Contents


**Clinical trial cost estimates ........ 2**

**Sources for trial cost data reported in Table 2 of the main text ........ 3**

**Estimation of revenues deriving from priority review voucher ........ 4**

**Product donations ........ 5**

**Estimates of originator clinical trial expenditures and the Orphan Drug Tax Credit ........ 7**

**Regulatory exclusivities ........ 9**

**US Food and Drug Administration and European Medicines Agency fees ........ 9**

**Requirements placed on Janssen by regulators as a condition of accelerated approval ........ 10**

**Currency conversion rates ........ 12**

**Proportion of trial costs considered attributable to the development of bedaquiline ........ 13**

**Cost of capital and risk adjustment ........ 15**

**Estimates of public expenditure on bedaquiline clinical trials – risk-adjustment and capitalization (US$ millions, 2018) ........ 16**

**Estimates of Janssen investments in bedaquiline development, risk-adjustment, and capitalization (US$ millions, 2018) ........ 17**

**"Apples to apples" comparison of estimated public and Janssen clinical trial expenditures (2018 US$ millions) ........ 18**

## Clinical trial cost estimates

As described in Methods, where study leads did not provide clinical trial costs, we assumed trial costs based on estimates for costs of clinical trials for anti-infectives published by Sertkaya et al. (Table).[1]

Data used in the Sertkaya et al. study were for clinical trials undertaken 2004-2012, with no inflation adjustment. We therefore inflation-adjusted the averages reported in that study from 2008 (midpoint of 2004-2012) to 2018, using the US implicit GDP deflator.

| | **Phase 1** | **Phase 2** | **Phase 3** |
|---|---|---|---|
| Average trial costs across **all therapeutic areas**, as reported in Sertkaya et al. | $4,000,000 | $13,000,000 | $20,000,000 |
| *Inflation adjusted from 2008 to 2018 USD* | $4,660,952 | $15,148,095 | $23,304,762 |
| Average trial costs for **anti-infectives**, as reported in Sertkaya et al. | $4,200,000 | $14,200,000 | $22,800,000 |
| *Inflation adjusted from 2008 to 2018 USD* | **$4,894,000** | **$16,546,381** | **$26,567,428** |

[1] Sertkaya A, Wong H-H, Jessup A, Beleche T. Key cost drivers of pharmaceutical clinical trials in the United States. *Clinical Trials* 2016; **13**: 117–26.

## Sources for trial cost data reported in Table 2 of the main text

| Short title of trial | Source for cost data |
|---|---|
| InDEX | Email from Nesri Padayatchi (Centre for the AIDS Programme of Research in South Africa; CAPRISA) to Dzintars Gotham on December 4, 2018 |
| NiX-TB-(B-L-Pa) | Did not respond to request for data by time of submission; estimated based on Sertkaya et al. |
| TMC207-CL002 | Did not respond to request for data by time of submission; estimated based on Sertkaya et al. |
| NC-005-(J-M-Pa-Z) | Did not respond to request for data by time of submission; estimated based on Sertkaya et al. |
| TMC207-CL001 | Did not respond to request for data by time of submission; estimated based on Sertkaya et al. |
| NC-003-(C-J-Pa-Z) | Did not respond to request for data by time of submission; estimated based on Sertkaya et al. |
| NC-001-(J-M-Pa-Z) | Did not respond to request for data by time of submission; estimated based on Sertkaya et al. |
| ZeNix (B-Pa-L) NC-007 | Did not respond to request for data by time of submission; estimated based on Sertkaya et al. |
| SimpliciTB (B-Pa-M-Z) NC-008 | Did not respond to request for data by time of submission; estimated based on Sertkaya et al. |
| TASK-002 | Email from Sharon Nachman to Mark Harrington (Treatment Action Group) on January 14, 2019 |
| STREAM Stage 2 | Email form YaDiul Mukadi (USAID) to Dzintars Gotham on August 6, 2019 |
| endTB interventional | Email from Joshua Bogus (Partners In Health) to Dzintars Gotham on February 6, 2019 |
| TB-PRACTECAL | Email from Bern-Thomas Nyang'wa (Médecins Sans Frontières) to Dzintars Gotham on January 30, 2019 |
| ACTG 5343 | Email from Kelly Dooley (Johns Hopkins University) to Dzintars Gotham on November 23, 2018 |
| ACTG 5267[C] | Email from Kelly Dooley (Johns Hopkins University) to Dzintars Gotham on November 23, 2018 |
| TMC207 +/- Rifabutin/Rifampin | Did not respond to request for data by time of submission; estimated based on Sertkaya et al. |
| IMPAACT 1108 | Email from Sharon Nachman (SUNY Stony Brook) to Mark Harrington (Treatment Action Group) on January 14, 2019 |
| IMPAACT 1025/1026s | Email from Sharon Nachman (SUNY Stony Brook) to Mark Harrington (Treatment Action Group) on January 14, 2019 |
| TRUNCATE-TB | Email from Nick Paton (National University of Singapore) to Dzintars Gotham on November 28, 2018 |
| NEXT | Email from Kheertan Dheda (University of Cape Town) to Dzintars Gotham on November 28, 2018 |
| endTB observational | Email from Joshua Bogus (Partners In Health) to Dzintars Gotham on February 6, 2018 |
| Janssen C211 | Cook-Scalise, Sarah (TB Alliance). Personal communication with: Lindsay McKenna (Treatment Action Group) on May 27, 2015.. |
| endTB-Q | Email from Joshua Bogus (Partners In Health) to Dzintars Gotham on February 6, 2018 |

All mentioned respondents consented to being cited as sourced for these data.

## Estimation of revenues deriving from priority review voucher

A simplified model published by Ridley and Régnier in 2016 allows estimation of the value of a PRV based on two parameters – acceleration of approval in months, and fifth-year sales of the product to which the PRV is applied, with the assumption that the product is the first or second-entrant to the relevant market.[2]

The application for guselkumab was considered filed in January 2017 and was approved 6 months later in July 2017.[3] The standard review time is 10 months.[4] The acceleration of approval for guselkumab deriving from applying the PRV was therefore 4 months.

Ridley and Régnier estimated that for a first- or second-entrant medicine. given approval acceleration of 4 months, the value of a PRV would be US$384 million if fifth–year sales were US$1·50 billion, and US$448 million if fifth-year sales were US$1·75 billion. Fifth-year (2022) sales of guselkumab are projected to be US$1·6 billion.[5]

Guselkumab was the second approved IL-23 inhibitor, after ustekinumab. For the purposes of identifying which entrant (first, second, third, etc.) guselkumab is, it debatable whether the relevant market is limited to IL-23 inhibitors or includes also IL-17 inhibitors and/or TNF-alpha inhibitors. In the latter case, guselkumab could be seen as the third or fourth entrant. For such 'late entrants', the value of the PRV is reduced by approximately a quarter (see Exhibit 2 in Ridley and Régnier). Therefore, based on this model, the value of the PRV to Janssen can be estimated to lie in the range US$300–400 million.

Ridley and Régnier model from which these figures were derived includes a number of other assumptions, all of which we considered to be broadly applicable to guselkumab.

As a sensitivity analysis, we calculated the net present value for an additional 4 months of sales at the level projected for guselkumab in its fifth year. With fifth-year (2022) sales, as previously mentioned, projected to be US$1·6 billion, 4 months of sales at this level would equal approximately US$533 million. Applying discounting of 10·5%/year (the level suggested in Ridley and Régnier) yields a net present value in 2018 of US$342 million – a value very similar to that given by the Ridley and Régnier model.

Using a PRV is associated with a fee of US$2·7 million in 2017, or US$2·8 million in 2018 US dollars. This amount could be subtracted from the estimated value of the PRV to account for 'added' costs to the applicant of using a PRV versus a normal application. However, as the PRV fee is dwarfed by the estimated PRV value, we leave this out, for simplicity.

---

[2] Ridley DB, Régnier SA. The Commercial Market For Priority Review Vouchers. *Health Affairs* 2016; **35**: 776–83.

[3] US Food & Drug Administration. Center for Drug Evaluation and Research. Application Number:761061orig1s000. Administrative And Correspondence Documents. Available from: https://www.accessdata.fda.gov/drugsatfda_docs/nda/2017/761061Orig1s000Admincorres.pdf and https://www.accessdata.fda.gov/drugsatfda_docs/appletter/2017/761061Orig1s000ltr.pdf

[4] US Food & Drug Administration. FY 2017 Performance Report To Congress for the Prescription Drug User Fee Act. Available from: https://www.fda.gov/downloads/AboutFDA/ReportsManualsForms/Reports/UserFeeReports/PerformanceReports/UCM606719.pdf

[5] EvaluatePharma® World Preview 2017, Outlook to 2022. 10th Edition – June 2017. Available from: http://info.evaluategroup.com/rs/607-YGS-364/images/WP17.pdf

## Product donations

We emphasise that the following is an academic exercise in estimating potentially tax-deductible amounts, based on limited publicly available information.

We take two approaches to estimating tax deductions from product donations. The first is based on the cost of manufacture for bedaquiline, as reported by a Janssen representative. The second is based on reports on charitable contributions published by Janssen.

*Estimate based on cost of manufacture*

The deductible expense is calculated as twice the cost of making the product (in this context termed the 'cost basis') or the midpoint between the 'cost basis' and the 'fair market value', whichever is lower.[6] Currently, the lowest selling price offered by Janssen for bedaquiline is US$400 per treatment course in South Africa,[7] and the lowest selling price prior to this was US$900 per treatment course.[8] A representative of Janssen recently noted that approximately a third of this price covers the cost of manufacture and distribution, a third covers regulatory costs and pharmacovigilance, and a third covers 'programmatic' expenses on such as appropriate use.[9] Based on these comments, we assume that Janssen reported a 'cost basis' for each donated course of bedaquiline of US$133, corresponding to the manufacturing and distribution costs. The actual 'cost basis' claimed may be higher, which would make the value of the tax deduction greater. We assumed that the claimed 'fair market value' of bedaquiline was greater than twice the cost basis, and that the deductible amount per donated treatment course was therefore US$266. Additionally, Janssen has published reports on their charitable donations of bedaquiline product to USAID. We derived a second estimate based on these reported donations.

The Janssen/USAID/GDF donation programme has (will have) donated 105,000 treatment courses of bedaquiline over 2015-2019.[10] For the purposes of inflation adjustment, we assumed the same amount of donation expenses are claimed in each year from 2015-2019, inclusive (Table).

The reduction in taxes deriving from a deductible expense can be calculated as the product of the deductible expense and the corporate tax rate.

| Year | Assumed number of donated treatment courses for which deductible expenses claimed | Assumed deductible expense claimed before inflation adjustment | Inflation adjustment factor (to 2018 USD) | Assumed deductible expense claimed after inflation adjustment | Reduction in tax bill* |
|---|---|---|---|---|---|
| 2015 | 21,000 | $5,586,000 | 1·04216102 | $5,821,511 | $2,037,529 |
| 2016 | 21,000 | $5,586,000 | 1·03098073 | $5,759,058 | $2,015,670 |
| 2017 | 21,000 | $5,586,000 | 1·01799317 | $5,686,510 | $1,990,278 |
| 2018 | 21,000 | $5,586,000 | 1 | $5,586,000 | $1,173,060 |
| 2019 | 21,000 | $5,586,000 | 0·98232486 | $5,487,267 | $1,152,326 |
| **Total** | **105,000** | **$27,930,000** | | **$28,340,346** | **$8,368,864** |

[6] 26 U.S. Code § 170 - Charitable, etc., contributions and gifts. https://www.law.cornell.edu/uscode/text/26/170 (accessed Jan 14, 2019); and Matthew Scaliti. Tax deduction for pharmaceutical drug inventory. RSM. 2018; published online Dec 7. https://rsmus.com/what-we-do/industries/life-sciences/tax-deduction-for-pharmaceutical-drug-inventory.html (accessed Dec 12, 2018)

[7] International Union Against Tuberculosis and Lung Disease. South Africa announces lower price for TB drug bedaquiline. 2018; published online July 23. https://theunion.org/news-centre/news/south-africa-announces-lower-price-for-tb-drug-bedaquiline (accessed May 22, 2019).

[8] MSF Access Campaign. DR-TB drugs under the microscope: Sources and prices for drug-resistant tuberculosis medicines. 4th edition. 2016 http://www.msfaccess.org/sites/default/files/TB_report__DR-TB_DRUGS_UTM__4th_edition_2016.pdf (accessed March 22, 2016).

[9] Statements by Adrian Thomas (Vice President, Access, Programs & Policy, Global Public Health, Johnson & Johnson) at the Private Sector Engagement on TB Solutions - SDG Media Zone at the 73rd session of United Nations General Assembly. 26 September 2018. Video, minutes 14:38-14:58. Available from: http://webtv.un.org/search/private-sector-engagement-on-tb-solutions-sdg-media-zone-at-the-73rd-session-of-united-nations-general-assembly/5840554073001/

[10] Stop TB Partnership. Global Drug Facility. GDF Bedaquiline Order Status Report. 06-March-2019. Available from: http://www.stoptb.org/assets/documents/gdf/GDF_BDQ_Rpt_06-Mar-2019.pdf

*Calculated by multiplying assumed deductible expense claimed after inflation adjustment by the corporate tax rate in the respective year: 35% for fiscal years 2015, 2016, and 2017, and 21% for fiscal years 2018 and 2019.

*Estimate based on reported charitable donations*

A second potential source for estimating tax deductions deriving from donations of bedaquiline stems from reported charitable contributions.

Janssen documents report donations of bedaquiline to USAID equivalent to 'payment amounts' of US$ 1,000,000 on 23 of October 2015, 500,000 on the 5th of August 2016, and US$ 29,310,000 to USAID on the 17th of December 2015 and US$ 44,580,000 on the 1st of December 2016.[11] The expected tax deductions, assuming deductions were claimed for these full amounts, and using inflation adjustment and corporate tax rates as in the Table below, would be US$27 million.

| Year | Assumed deductible expense claimed before inflation adjustment | Inflation adjustment factor (to 2018 USD) | Assumed deductible expense claimed after inflation adjustment | Reduction in tax bill* |
|---|---|---|---|---|
| 2015 | $29,310,000 | 1·04216102 | $30,545,739 | $10,691,009 |
| 2016 | $44,580,000 | 1·03098073 | $45,961,121 | $16,086,392 |
| **Total** | **$73,890,000** | N/A | **$76,506,860** | **$26,777,401** |

*Calculated by multiplying assumed deductible expense claimed after inflation adjustment by the corporate tax rate in the respective year: 35% for fiscal years 2015, 2016.

We consider this a conservative (low) estimate, as tax deductions could be higher if Janssen has claimed further product donations in addition to those shown in the Table above. For example, Janssen has reported additional 'product donations' to USAID, including one with a 'payment amount' of US$18,992,000 on the 29th of November 2018, but these do not specify bedaquiline.[12] Details on what proportion of these donations were in fact claimed as tax deductible are not public, however. In addition, Janssen is reported to have made bedaquiline donations after 2016, which are not included here.

*Costs to Janssen of the donation program*

Similar to estimates of tax savings, above, we estimate the costs to Janssen of donating bedaquiline both based on estimated cost of production, and on reported donation expenses.

For cost of production, we multiplied US$133 by the assumed number of donated treatment courses – 105,000. This yields US$13,965,000. The sum of donation expenses in publicly available Janssen documents, attributable to bedaquiline, is $76,506,860 (see Table above).

*Totals*

Total Janssen expenses for the donation programme are thus estimated at US$14-77 million, while tax savings from the program are estimated at US$8-27 million.

[11]Senate Committee on Finance Questions for the Record Drug Pricing in America: A Prescription for Change, Part II. February 26, 2019. Questions for: Jennifer Taubert, Executive Vice President, Worldwide Chairman, Janssen Pharmaceuticals Johnson & Johnson. Spending on Advertising/Marketing vs. Research and Development. Available from: https://www.finance.senate.gov/imo/media/doc/Johnson%20and%20Johnson%20Responses.pdf

[12] Janssen Therapeutics, division of Janssen Products, LP, Monetary and Product Contribution to US based Charitable Organizations. Full Year 2018 (Payments made from January 1, 2018 to December 31, 2018) - Amended 4.19.19. Available from: https://www.janssen.com/sites/www_janssen_com_usa/files/2018-jt-charitable-contribution-transparency-amended-4.19.19v2.pdf

## Estimates of originator clinical trial expenditures and the Orphan Drug Tax Credit

In the applicable time period, the Orphan Drug Act entitled pharmaceutical companies to a tax credit of up to 50% of qualifying research expenditures, between the date that orphan drug designation is granted (2005 for bedaquiline) and the date the drug is approved (2012 for bedaquiline in the US).[13] (Following a change in tax law, the credit has now been reduced to 25%.[14]) As described above, we used estimates of average clinical trial cost by phase to estimate Janssen's total clinical trial spending. As most trials spanned numerous years, we assumed these expenditures are evenly attributed across years and the ODTC is claimed on expenditures each year. Values were inflation-adjusted to 2018 US dollars using the US GDP deflator. The Act also requires that the relevant clinical studies have been done in the US, but an exception exists for where there would not be a sufficient number of trial participants in the US alone.[15] We assume that this exception was employed.

**Table. Calculation of estimated orphan drug tax credit.**

| | | | | **Assumed trial expenses allocated by year (US$ millions)** | | | | | | | |
|---|---|---|---|---|---|---|---|---|---|---|---|
| **Trial (short title)** | **Phase** | **Cost estimate[A]** | **Study duration** | **2005** | **2006** | **2007** | **2008** | **2009** | **2010** | **2011** | **2012** |
| TMC207-C202 | 2 | $16,546,381 | 2005–05 | $16·5 | $0·0 | $0·0 | $0·0 | $0·0 | $0·0 | $0·0 | $0·0 |
| TMC207-TiDP13-C208 | 2 | $16,546,381 | 2007–12 | $0·0 | $0·0 | $2·8 | $2·8 | $2·8 | $2·8 | $2·8 | $2·8 |
| TMC207-TiDP13-C110 | 1 | $4,894,000 | 2009–09 | $0·0 | $0·0 | $0·0 | $0·0 | $4·9 | $0·0 | $0·0 | $0·0 |
| TMC207-TiDP13-C117 | 1 | $4,894,000 | 2009–10 | $0·0 | $0·0 | $0·0 | $0·0 | $2·4 | $2·4 | $0·0 | $0·0 |
| TMC207-TiDP13-C209 | 2 | $16,546,381 | 2009–13 | $0·0 | $0·0 | $0·0 | $0·0 | $3·3 | $3·3 | $3·3 | $3·3 |
| TMC207-TIDP13-C111 | 1 | $4,894,000 | 2009–10 | $0·0 | $0·0 | $0·0 | $0·0 | $2·4 | $2·4 | $0·0 | $0·0 |
| TMC207-TiDP13-C112 | 1 | $4,894,000 | 2010–11 | $0·0 | $0·0 | $0·0 | $0·0 | $0·0 | $2·4 | $2·4 | $0·0 |
| TMC-207-TBC1003 | 1 | $4,894,000 | 2011–11 | $0·0 | $0·0 | $0·0 | $0·0 | $0·0 | $0·0 | $4·9 | $0·0 |
| TMC207TBC3001 | 2 | $8,273,190[B] | 2012–16 | $0·0 | $0·0 | $0·0 | $0·0 | $0·0 | $0·0 | $0·0 | $1·7 |
| TMC207TBC1002 | 1 | $4,894,000 | 2013–13 | $0·0 | $0·0 | $0·0 | $0·0 | $0·0 | $0·0 | $0·0 | $0·0 |
| TMC207TBC4001 | 4[C] | $26,567,428 | 2013–18 | $0·0 | $0·0 | $0·0 | $0·0 | $0·0 | $0·0 | $0·0 | $0·0 |
| TMC207-TIDP13-C210 | 3 | $0 (withdrawn) | 2014–22 | $0·0 | $0·0 | $0·0 | $0·0 | $0·0 | $0·0 | $0·0 | $0·0 |
| TMC207TBC2001 | 2 | $16,546,381 | 2015–20 | $0·0 | $0·0 | $0·0 | $0·0 | $0·0 | $0·0 | $0·0 | $0·0 |
| TMC207-TIDP59-C211 | 2 | $16,546,381 | 2016–25 | $0·0 | $0·0 | $0·0 | $0·0 | $0·0 | $0·0 | $0·0 | $0·0 |
| TMC207LEP2001 | 2 | $16,546,381 | 2018–21 | $0·0 | $0·0 | $0·0 | $0·0 | $0·0 | $0·0 | $0·0 | $0·0 |
| **Total assumed expenses 2005–2012** | | | | **$43–72 million[D]** | | | | | | | |
| **ODTC at 50%** | | | | **$22–36 million[D]** | | | | | | | |

Study Phases and duration gathered from ClinicalTrials.gov.
[A]Trial cost estimates from Sertkaya et al, inflation adjusted to 2018 US$.
[B]Assumed cost (average Phase 2 trial cost from Sertkaya et al) has been halved as this is an early-access programme rather than a traditional clinical trial.
[C]While for the other trials the Phases are part of the listing on ClinicalTrials.gov, in this case we have assumed that it would be classified as Phase 4.

---

[13] U.S. Congress. 26 USC §45C. Clinical testing expenses for certain drugs for rare diseases or conditions. 2013; published online Jan 15. http://uscode.house.gov/view.xhtml?hl=false&edition=2012&req=granuleid%3AUSC-2017-title26-section45C&num=0 (accessed Jan 9, 2019).
[14] U.S. Congress. PUBLIC LAW 115–97—DEC. 22, 2017. Sec. 13401. Modification of orphan drug credit. 2017; published online Dec 22. https://www.govinfo.gov/content/pkg/PLAW-115publ97/pdf/PLAW-115publ97.pdf (accessed Jan 14, 2019).
[15] U.S. Congress. 26 USC §45C. Clinical testing expenses for certain drugs for rare diseases or conditions. 2013; published online Jan 15. http://uscode.house.gov/view.xhtml?hl=false&edition=2012&req=granuleid%3AUSC-2017-title26-section45C&num=0 (accessed Jan 9, 2019).

[D]Range generated by reducing by 40% for lower limit, to account for potentially lower costs of trials run in LMICs.

### Regulatory exclusivities

Market exclusivity (ME) can translate to increased overall sales if it extends the length of monopoly rights beyond the period protected by patents. Orphan drug designation, which was granted to bedaquiline in the US and EU (see above), confers market exclusivity for the respective indication: 7 years from approval in the US and 10 years from approval in the EU (plus 2 years if agreed paediatric investigations have been undertaken).[16] Based on approval dates, this potentially means ME for the MDR-TB indication until 2019 in the US, and ME until 2025 in the EU. However, bedaquiline is protected by patents until, earliest, 2024.[17]

### US Food and Drug Administration and European Medicines Agency fees

We assumed that the US FDA new drug application (NDA) filing fee for bedaquiline was waived, as provided for medicines with orphan designation.[18] The NDA filing fee was US$1.8 million in 2012,[19] which is US$2.0 million in 2018 US dollars. The waiver of application fees for orphan-designated NDAs is arguably a subsidy from the FDA to the applicant and could be counted as a public contribution to bedaquiline development but roughly 'cancels out' with the fee for using a PRV.

The EMA offers reduced fees for products with orphan designation. The savings from reduced fees charged by the EMA due to orphan designation were estimated at EUR 46,840 (see blow). As this amount is negligible compared to other public contributions estimated here, we do not include it in calculations, for simplicity.

The application for bedaquiline was submitted to the EMA on 28 August 2012.[20] Therefore the fee schedule adopted on 23 August 2012 would have applied. We assume than Janssen was not considered a micro, small or medium sized enterprise.

| Item | Normal fee (EUR) | Reduction with orphan drug designation |
|---|---|---|
| Basic fee for application for marketing authorisation through centralised procedure | 267400 | –10% |
| Inspection (Level I) | 20100 | –100% |
| Maintenance fee (Level I) | 95900 annually | No applicable reduction for applicants not classed as micro, small or medium sized enterprises |
| Scientific advice / assistance in protocol development | To our knowledge, Janssen did not receive these services from EMA | |

Sources: EMA rate tables obtained through the 'access to documents' procedure and can be shared on request.

16 EvaluatePharma. Orphan Drug Report 2018. 2018; published online May. http://info.evaluategroup.com/rs/607-YGS-364/images/OD18.pdf (accessed Dec 11, 2018).
17 Unitaid. A Review of the Bedaquiline Patent Landscape: A scoping report. 2014 https://unitaid.eu/assets/TMC_207_Patent_Landscape.pdf (accessed Dec 11, 2018).
18 U.S. Food and Drug Administration. Prescription Drug User Fee Amendments. https://www.fda.gov/industry/fda-user-fee-programs/prescription-drug-user-fee-amendments (accessed Aug 1, 2019).
19 Page 45831, Federal Register Vol. 76, No. 147. https://www.govinfo.gov/content/pkg/FR-2011-08-01/pdf/2011-19332.pdf (accessed Jan 9, 2019).
20 European Medicines Agency. 19 December 2013. CHMP assessment report: SIRTURO. Available from: https://www.ema.europa.eu/en/documents/assessment-report/sirturo-epar-public-assessment-report_en.pdf

## Requirements placed on Janssen by regulators as a condition of accelerated approval

FDA

As a condition of accelerated approval, which was made based mainly on a Phase 2 trial, the FDA required Janssen to undertake a number of additional trials. These are given below, cited from the FDA approval letter, which is available from:
https://www.accessdata.fda.gov/drugsatfda_docs/appletter/2012/204384Orig1s000ltr.pdf

1988-001: Conduct a confirmatory randomized double blind placebo controlled multicenter Phase 3 trial in subjects with sputum smear-positive pulmonary multidrug resistant tuberculosis (MDR-TB). This trial should assess long term outcomes of failure or relapse or death at least 6 months after all MDR-TB treatment is completed.

1988-002: Develop a patient registry for bedaquiline-treated patients to assess incidence rates of serious adverse events, including death

1988-003: In order to inform PMR 1988-005, conduct a study to define the Quality Control ranges of bedaquiline for MDR-TB isolates using standard proportion methods.

1988-004: In order to inform PMR 1988-005, conduct a study to define the Quality Control ranges of bedaquiline for MDR-TB isolates using MIC methods.

1988-005: Conduct a prospective in vitro study over a five-year period after introduction of SIRTURO (bedaquiline) to the market to determine MICs of MDR-TB isolates to bedaquiline for the first 5 years from marketing.

1988-006: Conduct an in vitro study to characterize the potential of bedaquiline and M2 as a substrate, inhibitor or inducer of the OATP1B1 and OATP1B3 drug transporters.

1988-007: Conduct a drug interaction trial of bedaquiline and efavirenz to determine a safe and effective dose regimen of both drugs when they are co-administered in HIV co-infected MDR-TB patients. Alternatively, adequate data from a previously conducted drug interaction trial may be submitted.

No paediatric trials were required of Janssen as bedaquiline received orphan drug designation and was thus exempt of this requirement.

EMA

Adults

A confirmatory Phase III study to evaluate additional efficacy and safety data of bedaquiline in different treatment regimen compared to a regimen that does not include bedaquiline.

European Medicines Agency. 19 December 2013. CHMP assessment report. SIRTURO. Available from:
https://www.ema.europa.eu/en/documents/assessment-report/sirturo-epar-public-assessment-report_en.pdf

Paediatrics
Study 1: Development of an age appropriate formulation

Study 2: Juvenile toxicity study in rats

Study 3: Open-label, randomised, crossover study in healthy adult subjects to determine the relative bioavailability of bedaquiline (fumarate) (TMC207) as tablet (for adults) to an age appropriate formulation and to investigate the food effect of the selected paediatric formulation

Study 4: Open-label, multicenter, single arm study to evaluate the pharmacokinetics, safety, tolerability and anti-mycobacterial activity of TMC207 in combination with a background regimen (BR) of multi-drug resistant tuberculosis (MDR-TB) medications for the treatment of children and adolescents from birth to less than 18 years of

European Medicines Agency. European Medicines Agency decision P/0403/2018 of 19 December 2018. Available from: https://www.ema.europa.eu/en/documents/pip-decision/p/0403/2018-19- december-2018-acceptance-modification-agreed-paediatric-investigation-plan-bedaquiline_en.pdf

## Currency conversion rates

2018 annual averages provided by the US Internal Revenue Service were used for currency conversions, available from https://www.irs.gov/individuals/international-taxpayers/yearly-average-currency-exchange-rates

| Trial | Reported cost | Reported currency | Converted to USD |
|---|---|---|---|
| NEXT | 50,000,000 | South African rand | 3,771,308 |
| TRUNCATE-TB | 10,000,000 | Singapore dollars | 7,412,898 |
| TB-PRACTECAL | 6,800,000 | Euro | 8,018,868 |

## Proportion of trial costs considered attributable to the development of bedaquiline

Trials sorted by start year.

| Title | Other IDs | % attributed to bedaquiline | Rationale |
|---|---|---|---|
| Pharmacokinetic Study of Antiretroviral Drugs and Related Drugs During and After Pregnancy | IMPAACT 1025/1026s | 0% | This study includes a large number of HIV and TB medicines; proportion attributable to bedaquiline alone considered to be negligible. |
| Safety, Tolerability, and Effect of TMC207 and Efavirenz in Healthy Volunteers | ACTG 5267 | 100% | Bedaquiline is the key investigational drug in this study |
| Evaluation of Early Bactericidal Activity in Pulmonary Tuberculosis | TMC207-CL001 | 100% | Bedaquiline is the key investigational drug in this study |
| Evaluation of Early Bactericidal Activity in Pulmonary Tuberculosis With | JMPaZ, NC-001 | 50% | Bedaquiline and pretomanid are both primary investigational medicines in this study |
| PK Interaction Between Rifapentine or Rifampicin and a Single Dose of TMC207 in Healthy Subjects | TMC207-CL002 | 100% | Bedaquiline is the key investigational drug in this study |
| TMC207 +/- Rifabutin/Rifampin | TMC207 +/- Rifabutin/Rifampin | 100% | Bedaquiline is the key investigational drug in this study |
| Evaluation of Early Bactericidal Activity in Pulmonary Tuberculosis With Clofazimine (C)-TMC207 (J)-PA-824 (Pa)-Pyrazinamide (Z) | NC-003 | 50% | Bedaquiline and pretomanid are both primary investigational medicines in this study |
| A Phase 2 to Evaluate the Efficacy, Safety and Tolerability of Combinations of Bedaquiline, Moxifloxacin, PA-824 and Pyrazinamide in Adult Subjects With Drug-Sensitive or Multi Drug-Resistant Pulmonary Tuberculosis. | NC-005 | 50% | Bedaquiline and pretomanid are both primary investigational medicines in this study |
| A Phase 3 Study Assessing the Safety and Efficacy of Bedaquiline Plus PA-824 Plus Linezolid in Subjects With Drug Resistant Pulmonary Tuberculosis | BPaL | 50% | Bedaquiline and pretomanid are both primary investigational medicines in this study |
| An Open-label RCT to Evaluate a New Treatment Regimen for Patients With Multi-drug Resistant Tuberculosis | NEXT | 100% | Bedaquiline is the key investigational drug in this study |
| TASK-002: Bioequivalence of Bedaquiline 400mg Administered in Crushed Form Compared to Tablet Form in Healthy Male and Female Adults Under Fed Conditions (BDQ Crush Study) | TASK-002 | 100% | Bedaquiline is the key investigational drug in this study |
| Expand New Drugs for TB [endTB] | endTB observational | 50% | Bedaquiline and delamanid are both primary investigational medicines in this study |
| Evaluating Newly Approved Drugs for Multidrug-resistant TB | endTB interventional | 50% | Bedaquiline and delamanid are both primary investigational medicines in this study |
| Evaluating Newly Approved Drugs in Combination Regimens for Multidrug-Resistant TB With Fluoroquinolone Resistance | endTB-Q | 50% | Bedaquiline and delamanid are both primary investigational medicines in this study |
| Evaluating the Safety, Tolerability, and Pharmacokinetics of Bedaquiline and Delamanid, Alone and in Combination, For Drug-Resistant Pulmonary Tuberculosis | ACTG 5343 | 50% | Bedaquiline and delamanid are both primary investigational medicines in this study |
| The Evaluation of a Standard Treatment Regimen of Anti-tuberculosis Drugs for Patients With MDR-TB | STREAM | 50% | The study has two key areas of focus: the use of a shortened regimen without bedaquiline, and the use of shortened regimens including bedaquiline. Bedaquiline is included in two of the three experimental arms in this study. |
| Safety and Efficacy of Various Doses and Treatment Durations of Linezolid Plus Bedaquiline and Pretomanid in Participants With Pulmonary TB, XDR-TB, Pre- XDR-TB or Non-responsive/Intolerant MDR-TB | ZeNix | 50% | Bedaquiline and pretomanid are both primary investigational medicines in this study |

| | | | |
|---|---|---|---|
| Evaluating the Pharmacokinetics, Safety, and Tolerability of Bedaquiline in HIV-Infected and HIV-Uninfected Infants, Children, and Adolescents With Multidrug-Resistant Tuberculosis | IMPAACT 1108 | 100% | Bedaquiline is the key investigational drug in this study |
| Pragmatic Clinical Trial for a More Effective Concise and Less Toxic MDR-TB Treatment Regimen(s) | TB-PRACTECAL | 50% | Bedaquiline and pretomanid are both primary investigational medicines in this study |
| Trial to Evaluate the Efficacy, Safety and Tolerability of BPaMZ in Drug-Sensitive (DS-TB) Adult Patients and Drug-Resistant (DR-TB) Adult Patients | SimpliciTB | 50% | Bedaquiline and pretomanid are both primary investigational medicines in this study |
| Two-month Regimens Using Novel Combinations to Augment Treatment Effectiveness for Drug-sensitive Tuberculosis | TRUNCATE-TB | 33% | Bedaquiline is included in one of five study arms, with the other four arms comprising well-established older TB medicines |
| Pharmacokinetic Study to Evaluate Anti-mycobacterial Activity of TMC207 in Combination With Background Regimen (BR) of Multidrug Resistant Tuberculosis (MDR-TB) Medications for Treatment of Children/Adolescents Pulmonary MDR-TB | TMC207-TIDP59-C211 | 100% | Bedaquiline is the key investigational drug in this study |
| Population Pharmacokinetics of Anti-tuberculosis Drugs in Children With Tuberculosis | BCH_PPK003 | 0% | The focus on bedaquiline in this trial appears to be limited |

### Cost of capital and risk adjustment

Many analyses of pharmaceutical R&D investments incorporate 'cost of capital' (COC) and risk-adjustment of costs to account for failed drug candidates.

Cost of capital represents the returns that the investor could have gained if they had spent money on an alternative investment with equal risk. Put another way, it represents the 'opportunity cost' – what are the returns that the investor has forgone by investing in bedaquiline development instead of investing, for example, in an index fund. When cost of capital is included in a number quantifying the costs of an investment, these are termed 'capitalised' costs. The inclusion of COC in estimates of drug R&D expenditures is controversial,[21] as inclusion tends to substantially increase the estimates expenditures, and does not represent actual cash that has been spent on R&D.

However, for the purposes of comparing estimated public expenditures with the bedaquiline R&D expenditures reported by the originator, we estimated capitalised costs and risk-adjusted costs for both public expenditures and expenditures by Janssen.

A cost of capital of 10% annually was assumed.

In order to calculate capitalised values, it was necessary to make assumptions regarding the distribution of the total expenses over the study period: we assumed even distribution from the study start year to the study completion year, as reported in the ClinicalTrials.gov database.

For risk-adjustment, per-Phase risk values reported by Sertkaya et al. were used:

67% success rate in Phase 1
41% success rate in Phase 2
65% success rate in Phase 3
83% overall success rate at registration stage.

For Janssen expenditures, figures with the orphan drug tax credit (ODTC) offsetting clinical trial expenditures were calculated by multiplying assumed clinical trial expenditures by 50% for years up to and including in 2012 (see Methods).

[21] Avorn J. The $2.6 Billion Pill — Methodologic and Policy Considerations. *New England Journal of Medicine* 2015; **372**: 1877–9.

**Estimates of public expenditure on bedaquiline clinical trials – risk-adjustment and capitalization (US$ millions, 2018)**

| Proportional attribution of trial costs to bedaquiline? | 40% reduction in assumed cost of clinical trials, where cost not reported by PI*? | |
|---|---|---|
| | **No** | **Yes** |
| **No** | 279 (C: 328, C/R: 733) | 219 (C: 246, C/R: 527) |
| **Yes** | 155 (C: 194, C/R: 443) | 120 (C: 142, C/R: 312) |

First number shown is estimated out-of-pocked expenditure. C – capitalized. C/R – capitalized and risk-adjusted.
*40% reduction is applied only to trial costs where estimates from Sertkaya et al. were used, and not to trials where costs were directly reported.

<u>Out-of-pocket, capitalized, and risk-adjusted capitalized public expenditures **with** proportional attribution and 40% reduction in costs (i.e. lower-range cost estimates)</u>

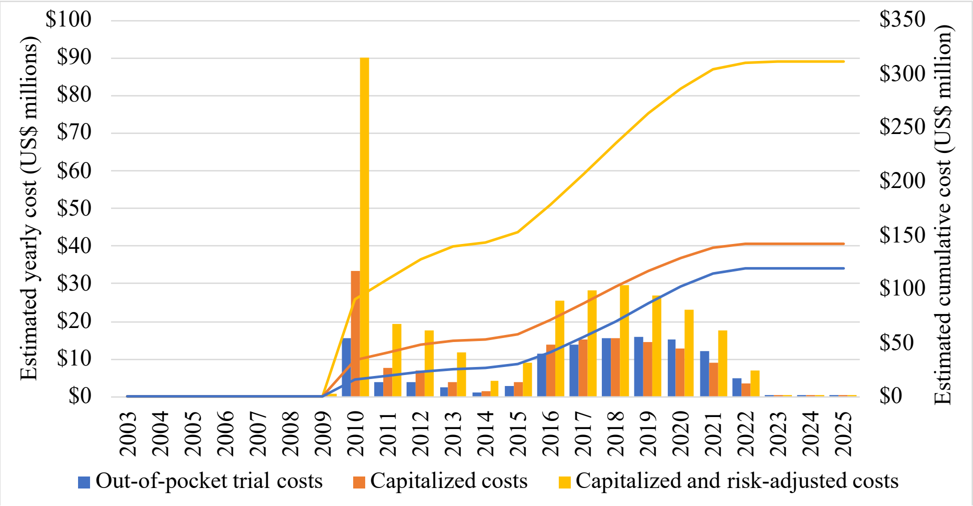


Note: expenditures 2003-2009 and 2023-2025 are not zero but are too low to be visible on this scale.

<u>Out-of-pocket, capitalized, and risk-adjusted capitalized public expenditures **without** proportional attribution and 40% reduction in costs (i.e. higher-range cost estimates)</u>

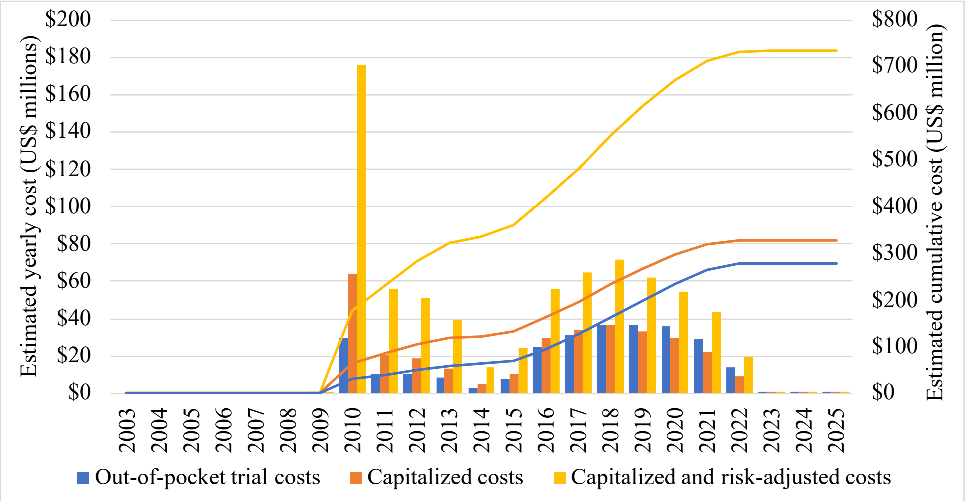


Note: expenditures 2003-2009 and 2023-2025 are not zero but are too low to be visible on this scale.

### Estimates of Janssen investments in bedaquiline development, risk-adjustment, and capitalization (US$ millions, 2018)

| Orphan drug tax credit offsetting clinical trial costs? | 40% reduction in assumed cost of clinical trials? | |
|---|---|---|
| | No | Yes |
| No | 163 (C: 280, C/R: 695) | 98 (C: 168, C/R: 417) |
| Yes | 127 (C: 191, C/R: 463) | 76 (C: 115, C/R: 278) |

First number shown is estimated out-of-pocked expenditure. C – capitalized. C/R – capitalized and risk-adjusted.

Out-of-pocket, capitalized, and risk-adjusted capitalized Janssen expenditures **with** 40% reduction in costs (i.e. lower-range cost estimate) and **with** ODTC offsetting clinical trial costs

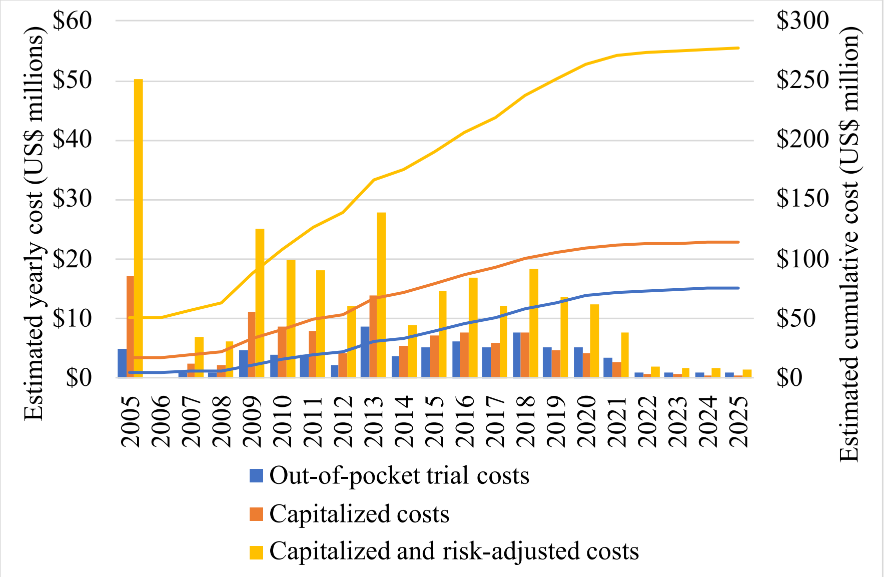


Out-of-pocket, capitalized, and risk-adjusted capitalized Janssen expenditures **without** 40% reduction in costs (i.e. higher-range cost estimate) and **without** ODTC offsetting clinical trial costs

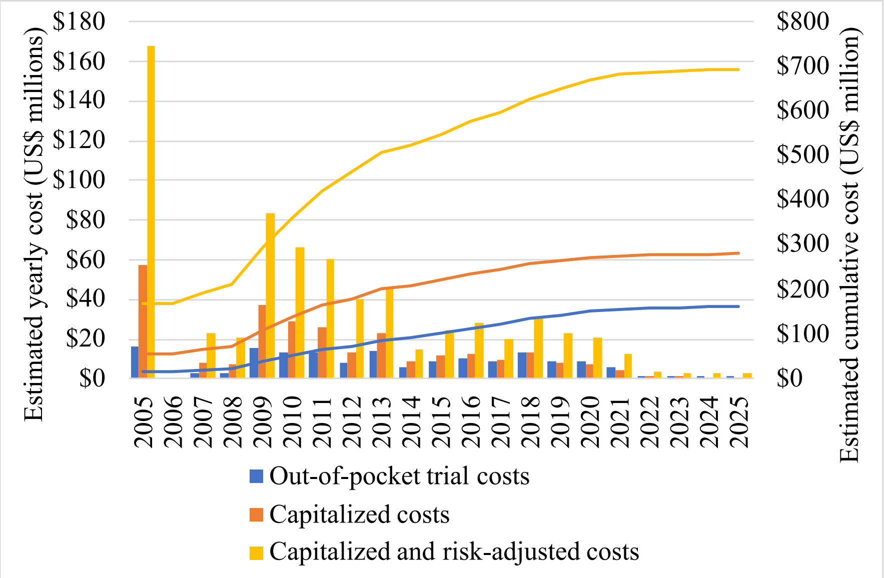

### “Apples to apples” comparison of estimated public and Janssen clinical trial expenditures (2018 US$ millions)

| Cost perspective | Public | Janssen |
|---|---|---|
| **Out of pocket** | 120-279 | 76-163 |
| **Capitalised** | 142-328 | 115-280 |
| **Capitalised and risk-adjusted** | 312-733 | 278-695 |

Ranges derive from whether or not 40% trial cost reduction is assumed, whether or not trial costs are offset by the ODTC (for Janssen), and whether whole trial costs are used or proportionally attributed to the bedaquiline component (for public expenditures; see above).
