# Supplementary material for: Public investments in the clinical development of bedaquiline
Source: PLoS One. 2020 Sep 18;15(9):e0239118. doi: 10.1371/journal.pone.0239118 (PMC7500616; doi:10.1371/journal.pone.0239118)
Supplement: Attachment — Submitted filename: TAG BDQ dev costs PLOS review response 2020-05-18_LM.docx [file pone.0239118.s003.docx]

**Responses to comments from editor and reviewers**

EDITOR COMMENT:

When submitting your revision, we need you to address these additional requirements.

1. Please ensure that your manuscript meets PLOS ONE's style requirements, including those for file naming. The PLOS ONE style templates can be found at
   <https://journals.plos.org/plosone/s/file?id=wjVg/PLOSOne_formatting_sample_main_body.pdf> and
   <https://journals.plos.org/plosone/s/file?id=ba62/PLOSOne_formatting_sample_title_authors_affiliations.pdf>

AUTHOR RESPONSE:

Thank you, the manuscript has been amended according to these requirements.

EDITOR COMMENT:

2. We note that you have indicated that data from this study are available upon request. PLOS only allows data to be available upon request if there are legal or ethical restrictions on sharing data publicly. For information on unacceptable data access restrictions, please see<http://journals.plos.org/plosone/s/data-availability#loc-unacceptable-data-access-restrictions>.

In your revised cover letter, please address the following prompts:

a) If there are ethical or legal restrictions on sharing a de-identified data set, please explain them in detail (e.g., data contain potentially identifying or sensitive patient information) and who has imposed them (e.g., an ethics committee). Please also provide contact information for a data access committee, ethics committee, or other institutional body to which data requests may be sent.

b) If there are no restrictions, please upload the minimal anonymized data set necessary to replicate your study findings as either Supporting Information files or to a stable, public repository and provide us with the relevant URLs, DOIs, or accession numbers. Please see <http://www.bmj.com/content/340/bmj.c181.long> for guidelines on how to de-identify and prepare clinical data for publication. For a list of acceptable repositories, please see <http://journals.plos.org/plosone/s/data-availability#loc-recommended-repositories>.

We will update your Data Availability statement on your behalf to reflect the information you provide.

AUTHOR RESPONSE:

Thank you, we have added the full dataset of (estimated) clinical trial cost, which would allow reproduction of our estimates, as a second Supplementary file.

EDITOR COMMENT:

3. Thank you for stating the following in the Financial Disclosure section:
"This analysis was funded by Treatment Action Group, New York (<http://www.treatmentactiongroup.org/>). There are no associated grant numbers. Three of the authors of this analysis are employees of TAG."
    
We note that one or more of the authors are employed by a commercial company: Independent, London, UK.

a)    Please provide an amended Funding Statement declaring this commercial affiliation, as well as a statement regarding the Role of Funders in your study. If the funding organization did not play a role in the study design, data collection and analysis, decision to publish, or preparation of the manuscript and only provided financial support in the form of authors' salaries and/or research materials, please review your statements relating to the author contributions, and ensure you have specifically and accurately indicated the role(s) that these authors had in your study. You can update author roles in the Author Contributions section of the online submission form.

Please also include the following statement within your amended Funding Statement.
“The funder provided support in the form of salaries for authors [insert relevant initials], but did not have any additional role in the study design, data collection and analysis, decision to publish, or preparation of the manuscript. The specific roles of these authors are articulated in the ‘author contributions’ section.”
If your commercial affiliation did play a role in your study, please state and explain this role within your updated Funding Statement.

AUTHOR RESPONSE:

Thank you, the affiliation for Dzintars Gotham – “Independent, London, UK.” – was intended to convey that he is a freelance researcher. Dzintars Gotham is not employed by any company/commercial entity, but he received funding from TAG for this research. We have changed DG’s affiliation to “Independent researcher, London, UK”, and have provided this updated Funding Statement in the cover letter.

"This analysis was funded by Treatment Action Group (TAG), New York (http://www.treatmentactiongroup.org/). There are no associated grant numbers. The funder provided support in the form of salaries for authors LM, MF, EL, and a grant to DG working as an independent researcher, but did not have any additional role in the study design, data collection and analysis, decision to publish, or preparation of the manuscript. The specific roles of these authors are articulated in the ‘author contributions’ section."

EDITOR COMMENT:

b) Please also provide an updated Competing Interests Statement declaring this commercial affiliation along with any other relevant declarations relating to employment, consultancy, patents, products in development, or marketed products, etc.

Within your Competing Interests Statement, please confirm that this commercial affiliation does not alter your adherence to all PLOS ONE policies on sharing data and materials by including the following statement: "This does not alter our adherence to  PLOS ONE policies on sharing data and materials.” (as detailed online in our guide for authors <http://journals.plos.org/plosone/s/competing-interests>) . If this adherence statement is not accurate and  there are restrictions on sharing of data and/or materials, please state these. Please note that we cannot proceed with consideration of your article until this information has been declared.

AUTHOR RESPONSE:

As explained above, Dzintars Gotham has no commercial affiliation. The Competing Interests Statement should thus remain: “The authors declare no potential conflicts of interest.”

Please include both an updated Funding Statement and Competing Interests Statement in your cover letter. We will change the online submission form on your behalf.

REVIEWER COMMENT:

Reviewer #1: The authors have clearly outlined the sources of R&D funding information, their approach to estimating data where requests for input were ignored, and the components of the R&D funding that have been included and excluded. The results are plainly and clearly presented. The limitations are also clearly identified and their potential scale and direction described.

My only suggestion is that, while the Union is now styled as such, to those not from the TB field, the name may be obscure. The previous full name - International Union Against Tuberculosis and Lung Disease - could be included in parentheses.

AUTHOR RESPONSE:

Thank you, we have added this in line 133, page 5.

REVIEWER COMMENT:

Reviewer #2: Congratulations to the authors for putting together such a concise, methodologically detailed and well thought out paper. I thoroughly enjoyed reviewing this paper as it provides a strong and critical argument around the considerations required when setting drug prices. Often R&D is cited as the reason behind the significant gaps between the estimated cost of production, as the and the market value of drugs, and therefore reflecting on public expenditure towards drug development in addition to that of pharmaceutical companies is relevant when negotiating prices for a public good. I wish the authors the best of luck with this paper moving forward.

I just have a few comments and points of clarity:

1. It would be good for some further explanation on how pharmaceutical companies set prices and the justification to set the price for Bedaquiline.

AUTHOR RESPONSE:

This is a challenging question, because pharmaceutical companies do not normally provide a justification for their pricing strategy, and pricing generally does not follow (for example) country average income level*. Where they have a monopoly, pharmaceutical companies generally set prices as high as the buyer will accept, as part of a profit-maximisation strategy.

However, bedaquiline is an exception, in that a company representative has in fact provided a justification for the current lowest price of ~ 400 USD. We have added in the company’s justification into the Discussion, lines 448-451, page 24 (see also answer to reviewer comment below).

*see for example:

1. Danzon PM, Furukawa MF. International Prices And Availability Of Pharmaceuticals In 2005. Health Aff (Millwood). 2008 Jan;27(1):221–33.
2. Danzon PM, Mulcahy AW, Towse AK. Pharmaceutical Pricing in Emerging Markets: Effects of Income, Competition, and Procurement: PHARMACEUTICAL PRICING IN EMERGING MARKETS. Health Econ. 2015 Feb;24(2):238–52.
3. Jayashree Watal, Rong Dai. Product Patents and Access to Innovative Medicines in a Post-TRIPS-Era. Staff Working Paper ERSD-2019-05. [Internet]. World Trade Organizaion. [cited 2020 Feb 26]. Available from: https://www.wto.org/english/res_e/reser_e/ersd201905_e.pdf
4. Scherer FM, Jayashree Watal. Post-TRIPS Options for Access to Patented Medicines in Developing Nations. J Int Econ Law. 2002 Dec 1;5(4):913–39.
5. Vogler S, Vitry A, Babar Z-U-D. Cancer drugs in 16 European countries, Australia, and New Zealand: a cross-country price comparison study. Lancet Oncol. 2016 Jan;17(1):39–47.
6. Sim J, Hill A. Is pricing of dolutegravir equitable? A comparative analysis of price and country income level in 52 countries. J Virus Erad. 2018 Oct 1;4(4):230–7.
7. Iyengar S, Tay-Teo K, Vogler S, Beyer P, Wiktor S, de Joncheere K, et al. Prices, costs, and affordability of new medicines for hepatitis C in 30 countries: an economic analysis. PLoS Med. 2016;13(5):e1002032.
8. Flynn S, Hollis A, Palmedo M. An economic justification for open access to essential medicine patents in developing countries. J Law Med Ethics. 2009;37(2):184–208.

REVIEWER COMMENT:

2. The authors reference that the cost of production of bedaquilin (lines 403) is $130, whilst Janssen has set the price of Bedaquilin as $400 to to some LMICs, while others pay $900.

AUHTOR RESPONSE:

Thank you – indeed there appears to be a gap between the lowest price offered by Janssen globally and the cost of production. In the same presentation by the Janssen representative that we reference for the quote that R&D investments were US$500 million, the representative stated that approximately a third of the US$400 price (~US$133) covers the cost of manufacture and distribution, one third covers regulatory and pharmacovigilance costs, and one third covers ‘programmatic’ expenses, such as for ensuring appropriate use.

We have added a note on this additional quote from Janssen on the breakdown of the 400 USD figure, in the Discussion, lines 448-451, page 24. This discussion had previously already appeared in the Appendix in the context of tax deductions for donated product.

REVIEWER COMMENT:

3. The authors assume that clinical trial costs in LMICs are assumed to be 40% lower. This is based on a reference comparing the costs in Asia to that of the US and European countries. Can the same be assumed for Africa?

AUTHOR RESPONSE:

This is a very generalised assumption for the comparison of clinical trial costs across different countries. To our knowledge, more precise quantitative estimates are not available. Indeed the 40% figure is a rough estimate in the source cited, and is given for China and India* (Frost & Sullivan, 2016).

It is very hard to say whether trial costs in Africa (or e.g. Russia, South America), would be expected to be of similar cost to the “40% lower” estimate for Asia. Bedaquiline trials included in our analysis have taken place all over the world (but nearly all in low- and middle-income countries). Originator trials took place in Russia, China, Ukraine, South Africa, India, Thailand (among others). Public sector trials are also taking place in a wide range of countries: e.g. TRUNCATE-TB (Philippines, Thailand, Singapore), TB Alliance trials (e.g. South Africa, Uganda, Tanzania, Russia, Georgia…), endTB (Georgia, Kazakhstan, Kyrgyzstan, Peru, South Africa, Lesotho).

We have added this a clarification in the relevant part of Methods to note that this 40% figure is extrapolated from China/India (lines 172-173, page 6).

*the exact quote from the source is “Asia offers lower costs for clinical trials. For example, costs in China and India are 25% to 40% lower than that of Western countries.” (including the 25% ‘tier’ would be meaningless as it would be ‘lost’ within the range).

REVIEWER COMMENT:

4. The authors reference a speech during the 2018 UN-High Level Meeting on Tuberculosis for the estimate of $500 million on R&D expenditures. Do the authors have any other reference for this estimate or a potentially be able to provide a time for the statement as the video is lengthy.

AUTHOR RESPONSE:

Thank you for this point – of course including a time stamp (if allowed by PLOS referencing style) will be helpful to readers.

The time stamp for these statements is 21:48–22:03. We have added this into the reference on lines 529, page 29. We have also added a more precise timestamp to footnote 9 on page 5 of the Appendix, which also refers to statements by the Janssen representative at the same event.

REVIEWER COMMENT:

5. In the interest of being as objective as possible, it is unlikely that the estimates provided by Janssen would not include costs that are risk adjusted and capitalized and therefore it is more likely that the $500 million estimate by Janssen can be substantiated. Furthermore, I feel that the authors should emphasize that the main finding is that public sector and originator clinical trial expenditures were similar-“comparing apples to apples” as the authors note, would be the most appropriate approach. That still however provides a strong argument for pricing to take into consideration that public sector investment and not allow for a ‘business as usual’ approach by the pharmaceutical company. Should pricing be in place to compensate Janssen for their R&D investments and stimulate further innovation, then the public sector should enjoy the same returns on their investments to stimulate further partnerships with the pharmaceutical industry.

AUTHOR RESPONSE:

We agree with the reviewer that Janssen’s stated R&D costs of $500 million are likely risk-adjusted and capitalized, which we explain in the Discussion (lines 404-410, page 22), and we do not intend to call into question Janssen’s stated costs, but simply to explain that it is likely a risk-adjusted and capitalized figure.

We have added to the first paragraph of the Discussion to prioritize the comparison of clinical trial costs before comparison of the overall public investments to originator costs. However, a key aim of our paper was to bring together these various ‘routes’ of R&D investment – not only clinical trial costs – so it is important to compare the ‘total’ public investments to originator investments.

We feel that it is important, in the ‘apples to apples’ comparison, always to present both the out-of-pocket cost as well as the capitalized/risk-adjusted cost (rather than prioritizing one over the other), as there has been extensive and robust debate over which cost is more representative. For example, many commentators* feel that risk-adjusted/capitalized numbers may be misleading to readers who are not used to using these accounting concepts, and will interpret these numbers as the out-of-pocket cost. (In addition, our assumption for cost of capital at 10.5%/annuum is perhaps on the high side, Prasad and Mailankody 2017, below.)

*See for example:

Avorn J. The $2.6 Billion Pill ? Methodologic and Policy Considerations. *New England Journal of Medicine* 2015; **372**: 1877–1879.

Light DW, Warburton R. Demythologizing the high costs of pharmaceutical research. *BioSocieties* 2011; **6**: 34–50.

Light DW, Lexchin JR. Pharmaceutical research and development: what do we get for all that money? *BMJ* 2012; **345**: e4348–e4348.

Prasad V, Mailankody S. Research and Development Spending to Bring a Single Cancer Drug to Market and Revenues After Approval. *JAMA Internal Medicine* 2017; **177**: 1569.

Love J. Perspectives on Cancer Drug Development Costs in JAMA. Bill of Health. Harvard Law School Petrie-Flom Center. 2017 Sep 13. Available from: https://blog.petrieflom.law.harvard.edu/2017/09/13/perspectives-on-cancer-drug-development-costs-in-jama/

REVIEWER COMMENT:

6.Some of the ratios of public to originator expenditures appear incorrect based on the methodology outlined on table 1. I would recheck all of the numbers

e.g

Capitalized clinical costs:
142/115=1.2
328/280=1.2

AUTHOR RESPONSE:

Thank you for catching this error. We have checked the ratios in the table and corrected these errors (page 12, Table 1).

EDITOR COMMENT:

While revising your submission, please upload your figure files to the Preflight Analysis and Conversion Engine (PACE) digital diagnostic tool, <https://pacev2.apexcovantage.com/>. PACE helps ensure that figures meet PLOS requirements. To use PACE, you must first register as a user. Registration is free. Then, login and navigate to the UPLOAD tab, where you will find detailed instructions on how to use the tool. If you encounter any issues or have any questions when using PACE, please email us at [figures@plos.org](mailto:figures@plos.org). Please note that Supporting Information files do not need this step.

AUTHOR RESPONSE:

Thank you, we are uploading PACE-approved Figure files.

OTHER CHANGES MADE:

We propose a replacement Figure 1, which we have tried to make visually more appealing, and have added estimated private expenditures for comparison.

Removed ‘claimed’ line 188, page 7 – not needed.

Heading changed to ‘Methods’ on line 129, page 5.

Updated number of TB deaths in introduction section to reflect 2018 estimates (line 148, page 6).

In the Discussion, lines 420-427, page 23, we compare our estimates for costs of bedaquiline R&D to the costs of R&D reported by the non-profit drug developer Drugs for Neglected Diseases initiative (DNDi). DNDi has recently published new estimates of their R&D costs, and we have therefore updated the relevant lines with new numbers, and updated the reference. The figures are similar to the earlier ones and do not affect the argumentation in the Discussion.

Reference #16 – link no longer worked, replaced with a new, working link.
